# Supplementary material for: An in vivo strategy for knockdown of circular RNAs
Source: Cell Discov. 2020 Aug 11;6:52. doi: 10.1038/s41421-020-0182-y (PMC7417560; doi:10.1038/s41421-020-0182-y)
Supplement: Supplementary file 1 — Supplementary Information [file 41421_2020_182_MOESM1_ESM.pdf]

Fig. S1

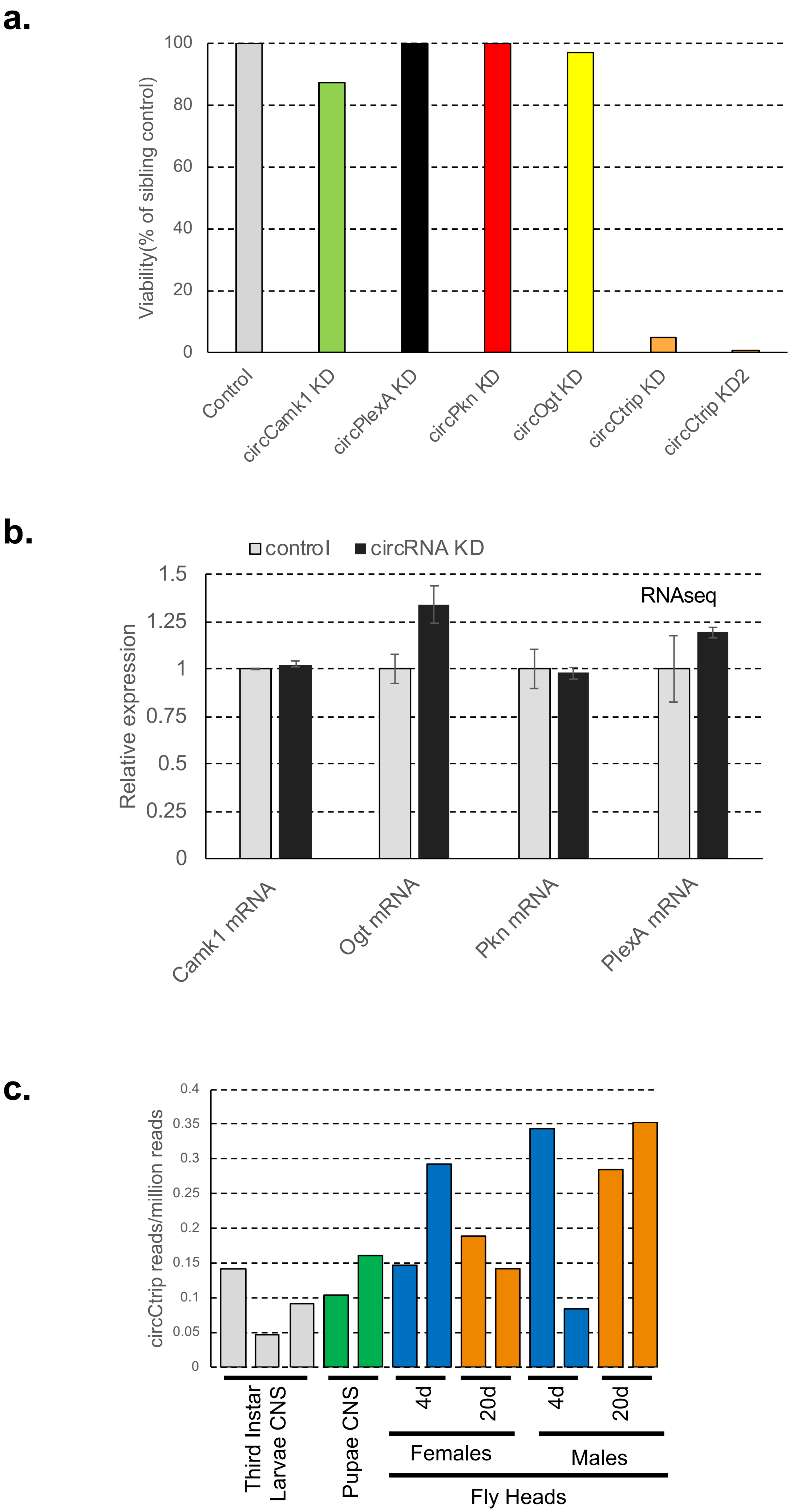

a.
Viability(% of sibling control)
100
80
60
40
20
0
Control
circCamk1 KD
circPlexA KD
circPkn KD
circOgt KD
circCtrip KD
circCtrip KD2
b.
control
circRNA KD
RNAseq
Relative expression
1.5
1.25
1
0.75
0.5
0.25
0
Camk1 mRNA
Ogt mRNA
Pkn mRNA
PlexA mRNA
c.
circCtrip reads/million reads
0.4
0.35
0.3
0.25
0.2
0.15
0.1
0.05
0
Third Instar Larvae CNS
Pupae CNS
4d
20d
4d
20d
Females
Males
Fly Heads

Legend for Supplementary Figure S1. a. Viability of all the circRNA knockdown strain compared to their sibling controls. For each KD strain we normalized the data to Cyo siblings (In all cases the experiment was performed at least three times). **b.** Relative levels of indicated mRNAs from 3`RNAseq data in their respective KD strains. Levels were normalized to mean expression in control line (*actin-Gal4* flies). In all cases we utilized the *actin-Gal4* flies as a control strain (n=3, error bars represent standard error of the mean). **c.** Expression of circCtrip at different developmental stages**.** Data was extracted from [18] and reanalyzed for circRNA expression. The Y axis indicates the number of circRNA reads per million reads.

Titles of Supplementary Tables and Movie

Supplementary Table S1. Oligonucleotides used in this study.

Supplementary Table S2. Normalized gene expression for the circRNA knockdown lines.

Supplementary Table S3. List of possible off-target genes and their expression changes in the different circRNA knockdown strains.

Supplementary Table S4. Genes differentially expressed upon knockdown of specific circRNAs.

Supplementary Table S5. Gene Ontology (GO) term analysis of the differentially expressed genes upon specific circRNA knockdowns.

Supplementary Table S6. Normalized gene expression for the circRNA knockdown lines under control of *elav-gal4* driver.

Supplementary Table S7. List of possible off-target genes and their expression changes in the two circCtrip KD strains.

Supplementary Table S8. Genes differentially expressed upon knockdown of specific circRNAs.

Supplementary Table S9. Gene Ontology (GO) term analysis of the differentially expressed genes upon circCtrip knockdowns.

Supplementary Movie S1. circCtrip KD escapers have impaired locomotion than control flies.
